# Effect of Intermittent Fasting on Immune Parameters and Intestinal Inflammation

**DOI:** 10.3390/nu16223956

**Published:** 2024-11-19

**Authors:** Eva Haasis, Anna Bettenburg, Axel Lorentz

**Affiliations:** Institute of Nutritional Medicine, University of Hohenheim, 70599 Stuttgart, Germany

**Keywords:** intermittent fasting, time-restricted feeding, alternate day fasting, inflammation, colitis, immune parameters

## Abstract

Intermittent fasting (IF), including alternate day fasting (ADF) and time-restricted feeding (TRF) or, in humans, time-restricted eating (TRE), has been associated with the prevention and improvement of diseases, including inflammatory bowel disease (IBD). This review summarizes 20 animal and human studies on the influence of IF on intestinal inflammation. In the animal studies, TRF and ADF improved histological scores, inflammatory markers, markers of oxidative stress, and microbiota composition. Apart from the studies on Ramadan fasting, there are no studies on IF in IBD patients, so human studies on IF in healthy people were included. The studies on Ramadan fasting showed almost no effects, but this particular type of fasting is not directly comparable to TRE or ADF. However, TRE and ADF appear to have anti-inflammatory effects in healthy individuals, as they significantly reduce CRP levels and inflammatory markers. TRE also improved the composition of microbiota and the circadian oscillation of clock genes. The beneficial effects of TRE and ADF in healthy people appear to depend on the number of uninterrupted days of fasting, while in animal studies improvements in colitis have been observed regardless of the duration of fasting.

## 1. Introduction

Intermittent fasting (IF) is a generic term for the dietary patterns that involve abstaining from food for certain periods, either days or hours [1]. This includes the dietary pattern known as “time-restricted feeding” (TRF) or, in humans, “time-restricted eating” (TRE), which involves a fasting period of more than ten hours between meals [2,3]. Typically, the fasting period ranges between 12 and 18 h [3,4,5,6]. An additional form of IF is alternate day fasting (ADF). In ADF, fasting days alternate with non-fasting days [2]. Other forms of IF are fasting twice-per-week (two consecutive or non-consecutive days per week) and fasting once-per-week [7]. Less common is long-term fasting, in which little or no food is consumed for several days or weeks [8]. Long-term fasting decreased intestinal permeability [9] and the concentration of pro-inflammatory cytokines such as the tumor necrosis factor (TNF)-α [10], both of which are involved in inflammatory bowel disease (IBD) [11]. In general, there are many combinations of fasting that can constitute a valid program based on the differences in the duration of continuous fasting and frequency of repeated fasting [10].

**Figure 1:**
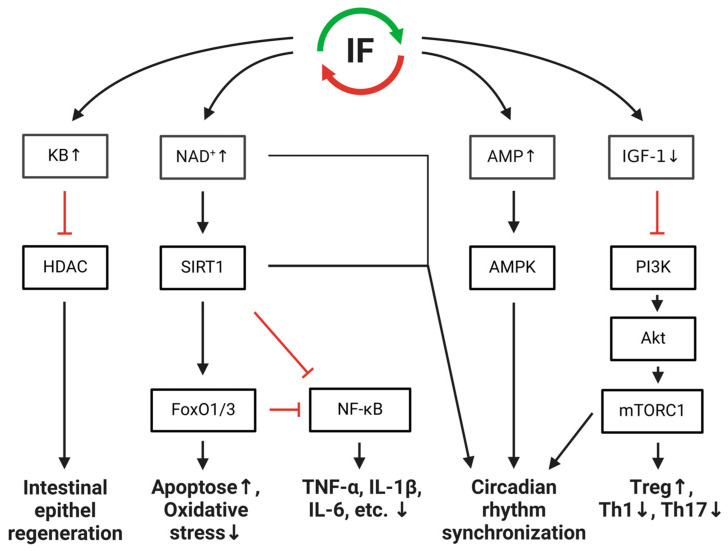
Effects of fasting on inflammatory signaling intermittent fasting (IF) increases the level of ketone bodies (KB) and inhibits histone deacetylases (HDACs), which promotes the regeneration of the intestinal epithelium. IF also lowers the plasma levels of insulin-like growth factor 1 (IGF-1) and downregulates phosphoinositide 3-kinase (PI3K)/Akt/mammalian target of rapamycin (mTOR) signaling pathways. There is an increase in regulatory T cells (Treg) and a reduction in effector T cells, such as T helper cells 1 (Th1) and Th17 cells. IF activates sirtuin 1 (SIRT1) and adenosine monophosphate-dependent kinase (AMPK) through the increase in nicotinamide adenine dinucleotide (NAD^+^) and AMP. NAD^+^, SIRT1, and AMPK can synchronize a circadian rhythm. SIRT1 and forkhead-box O 1/3 (FoxO1/3) inhibit nuclear factor kappa B (NF-κB). The inhibition of NF-κB downregulates pro-inflammatory cytokines such as the tumor necrosis factor α (TNF-α) and interleukin 1β (IL-1β). ↑ = upregulation; ↓ = downregulation. The figure was designed with BioRender (https://www.biorender.com; accessed on 27 September 2024).

IF has been associated with the prevention and improvement of diseases [12], including IBD [13]. The molecular mechanisms behind the health-promoting aspects can be attributed to an altered energy metabolism that occurs during fasting. Approximately twelve hours after the end of food intake, the liver’s glycogen stores are exhausted and fatty acids are metabolized to generate energy [14,15]. The result is a change in the availability or production of signal mediators or enzymes/transcription factors. Figure 1 summarizes the effects of fasting on inflammatory signaling pathways. For example, there is an increase in ketone body production, resulting in an increased expression of histone deacetylases (HDACs) inhibitors [16]. HDACs are involved in many inflammatory signaling pathways and can negatively affect the intestinal barrier [17]. In mouse studies, HDAC inhibitors improved intestinal inflammation [18,19,20]. Furthermore, fasting lowers insulin-like growth factor 1 (IGF-1) plasma levels [21,22] which downregulate the phosphoinositide 3-kinases (PI3K)/Akt/mammalian target of rapamycin (mTOR) signaling pathways [23,24]. The downregulation of the PI3K/Akt/mTOR signaling pathways leads to an increase in regulatory T cells and a reduction in effector T cells such as Th1- and Th17 cells [24], which can produce large amounts of pro-inflammatory cytokines [25]. In addition, fasting increases nicotinamide (NAD)^+^ levels. The intracellular NAD^+^ elevations are recognized by sirtuins and the forkhead-protein o (FOXO), activating them and inhibiting the transcription factor nuclear factor kappa B (NF-κB), followed by the downregulation of pro-inflammatory cytokines such as TNF-α and interleukin (IL)-1β [24,26,27,28,29].

Moreover, studies provide evidence that IF influences circadian rhythms. Mammals have developed an endogenous circadian clock that responds to the environmental light–dark cycle. In addition to the external light–dark cycle, meal timing and feeding rhythms are dominant Zeitgebers and can reset a circadian clock [23]. NAD^+^, sirtuins, and the AMP-activated protein kinase (AMPK), whose levels or activity are altered by fasting, influence circadian rhythms [24,25,26,27,28]. Fasting has been shown to improve the amplitude and stability of a circadian rhythm and synchronizes the oscillation phases [25,29]. A disruption of the clock has been implicated in diseases such as IBD. A disturbed circadian rhythm increases the susceptibility and severity of IBD [30]. First hints exist suggesting that fasting can restore intestinal clock functions and thus influence the severity of inflammatory diseases such as IBD [31].

The incidence and prevalence of IBD is increasing worldwide [32]. IBD affects the entire population, including children [33,34], adults [35,36], and the elderly [37,38]. Worldwide, around 6 to 8 million people are currently affected by a diagnosed chronic IBD, including 2 million in Europe and 1.5 million in North America [39]. IBD is a class of multifaceted chronic inflammatory gut disorders. The main types of IBDs are Crohn’s disease (CD) and ulcerative colitis (UC) [40]. They are characterized by uncontrolled inflammation that leads to intestinal damage [41].

The pathogenesis of IBD is multifactorial. In IBD, there is a disruption of the intestinal barrier [42]. This leads to a loss of intestinal epithelial cell integrity, with impaired tight junction function and a loss of the mucus layer, with reduced expression of antimicrobial peptides [41,42,43]. The disturbed intestinal barrier increases epithelial permeability and bacterial entry [11,44]. As a result, the cells of the innate immune system recognize the bacterial components and are activated [44]. This leads to the activation of NF-κB, resulting in an increased expression of pro-inflammatory cytokines and chemokines [45]. Effector cells are recruited and activated, leading to an imbalance between the regulatory T cells and effector T cells [11,44]. Ultimately, IBD leads to an inflammatory cycle [11,44]. In addition, IBD has been associated with disrupted circadian rhythms [46,47]. IBD and circadian rhythms influence each other. On the one hand, IBD leads to a disrupted rhythm, and on the other hand, a disrupted rhythm increases the severity of IBD [30]. The therapy for IBD involves immunosuppressive drugs [48,49,50]. However, some of these drugs cause undesirable side effects as infections, possible malignancies, pancreatitis, and morbidity factors [51,52,53,54,55]. Therefore, it is advisable to consider nonpharmacological strategies such as nutritional interventions as direct or adjunctive therapies for patients with IBD. Here, we provide an overview of studies on the influence of IF, specifically TRF and ADF, on intestinal inflammation.

## 2. Methods

The PubMed and Google Scholar databases were searched with the search terms “intermittent fasting”, “fasting”, “restrictive diet”, “time restricted feeding”, “TRF”, “alternate day fasting”, “ADF”, alone or in combination with “inflammatory bowel disease”, “IBD”, “intestinal inflammation”, “Crohn’s disease”, “ulcerative colitis”, “colitis”, “dextran sulfate sodium”, and “DSS” for relevant studies on the effect of IF on IBD in humans and on intestinal inflammation in animal models. Only the animal models using chemically induced colitis were considered. As there were no studies investigating IF in IBD patients, with the exception of Ramadan fasting, the existing studies on the effects of IF on immune parameters in healthy individuals were included using the additional search terms “human(s)”, “health(y)”, “men”, and “women” in combination with the above-mentioned search terms concerning fasting.

## 3. Effect of IF on Colitis in Animal Models

Fasting effects on chemically induced colitis have been studied in mice [56,57,58,59,60,61] and rats [62,63]. Colitis was induced by treatment with dextran sulfate sodium (DSS) in the mice [56,57,58,59,60,61] or acetic acid (AA) in the rats [62,63]. The intermittent fasting types were one-time fasting [56,57], TRF [58,59,62,63], and ADF [59,60,61]. Table 1 summarizes the fasting effects in the colitis models of mice and rats.

One-time fasting, no matter how long and during or after DSS treatment, seems to ameliorate inflammation in colon tissue [56,57]. A total of 48 h of fasting significantly reduced the inflammatory markers IL-1β and IGF-1, as well as the colitis activity score for the colon tissue of the mice that received 5% DSS for 5 days where fasting occurred the last 2 days [56]. Similarly, the mice that received 3.5% DSS for 5 days and whose food has been removed for 36 h after the administration of DSS showed a significant reduction in mRNA expression for cytokines IL-1β and IL-17. Moreover, their crypt numbers were increased by fasting [57].

Four studies analyzed the different types of TRF in rats and mice [58,59,62,63]. The measurement of TNF-α and glutathione (GSH) levels in the blood serum revealed significantly reduced serum levels in the female Wistar rats that had access to food for 2.5 h a day after colitis was induced via AA [62]. Fasting for 12, 16, or 20 h for 21 days was analyzed in Sprague Dawley rats after colitis induction with AA. Fasting significantly reduced the histological scores of the colon tissue, regardless of how long the fast lasted. Similar observations were made for the serum levels of IL-1β, IL-6, and IL-8, which were significantly ameliorated through any kind of fasting. In the liver, GSH was increased due to fasting. Colitis and oxidative damage seemed to be ameliorated by fasting, regardless of how long the fast lasted [63]. To explore the question whether TRF can improve colitis, another study was performed in which mice were given 2.5% DSS for five days, followed by water for 9 days in three repeated cycles. During the second and third cycles, the mice fasted for 18 h a day for 7 days. The disease activity index (DAI) scores significantly decreased in the TRF groups. The number of crypts increased and histological scores for the colon tissue were significantly decreased in the TRF groups compared to the control group. The percentage of CD4^+^ T cells in peripheral blood was significantly decreased by TRF, as well as the percentage of CD8^+^ T cells, compared to the control group. CD4^+^ T cells in mesenteric lymph nodes decreased significantly in the TRF group. In the TRF groups, the percentage of CD4^+^ and CD25^+^ T cells in the mesenteric lymph nodes were significantly increased compared to the control group. TRF significantly ameliorated the infiltration of leukocytes and macrophages around the crypt base in the lamina propria of the colon [58].

A total of 16 h of fasting a day were examined in mice that received 2% DSS during TRF treatment. The disease activity index was significantly decreased in the TRF group compared to the ad libitum (AL) group; additionally, the colon length improved, and goblet cell numbers significantly increased. The histological scores of the colon tissues were significantly ameliorated by TRF. The gene expression of the inflammatory markers TNF-α, IL-1β, IL-6, and IFN-γ as well as the TNF-α and IL-1β protein levels in the colon tissues of the TRF group were significantly enhanced compared to the DSS group. Claudin-1, zonula occludin (ZO)-1, occludin, and mucin2 (MUC-2) were significantly elevated compared to the DSS group. In addition, oxidative markers and microbiota composition were investigated. Oxidized glutathione (GSSG) significantly declined and GSH increased in the cortex tissue. The influence of TRF on microbiomes was only investigated in this study. TRF significantly suppressed the enrichment of the colitis-associated bacteria Gammaproteobacteria and Enterobacteriaceae in the colitis mice at the genus level. The levels of Escherichia coli and Shigella were inhibited by TRF in the mice that received DSS. The enrichment of Escherichia coli and Shigella were associated with colitis. The short chain fatty acid-producing microbes Rikenellaceae, Lactobacillus, Coproccus, and Ruminococcus as well as their products acetate, butyrate, and isobutyrate were significantly enhanced by TRF [59]. Taken together, this shows that time-restricted feeding improves inflammation in colitis, regardless of the number of hours of fasting.

Two studies analyzed ADF in combination with 2% DSS after fasting [60,61]. ADF significantly improved the histological scores concerning inflammatory cell infiltration, loss of goblet cells, damage of crypts, and mucosal destruction compared to ad libitum feeding. The DAI scores were significantly improved in the ADF group compared to the AL group, including colon length. Moreover, IL-1α, IL-6, KC, and G-CSF were significantly decreased in the ADF group compared to the AL group. ADF had a larger influence on histological scores than on inflammatory markers [60]. Regarding the colon, ADF had a strong bias for DSS-induced colitis. In jejunum, ADF significantly reduced occludin levels compared to the DSS group. ADF strongly ameliorated gut barrier function. The dietary therapy was started 6 weeks after the beginning of the DSS treatment (0.5%) and lasted 4 weeks. The relative abundance of Roseburia spp. and Bacteroides fragilis were enhanced by ADF. Meanwhile, the abundance of Roseburia spp. was decreased by DSS. ADF inverted the influence of DSS for Firmicutes and Bacteroides. Regarding bile acids, the impact of ADF was stronger. In addition, ADF improved barrier function in the small intestine, as well as the relative abundance of colitis-associated bacteria [61].

In the animal studies, fasting, no matter how many hours it lasted and whether or not it was repeated, improved inflammation in the colitis and the relative abundance of colitis-associated bacteria. Except for Ramadan fasting, there are no studies that have investigated fasting in IBD patients. A possible reason for this could be that many IBD patients avoid certain foods. In one study, around two thirds of IBD patients reported partial or complete avoidance of at least one food category [64].

## 4. Effect of Ramadan Fasting on IBD in Humans

The studies on Ramadan fasting were focused on tolerance or potential negative effects on IBD patients. During Ramadan, Muslims fast by abstaining from food and drink from sunrise to sunset. The effect of Ramadan fasting on IBD was examined in three human studies [65,66,67], including 60 IBD (43 UC, 17 CD) [65], 100 CD [66], and 80 IBD (60 UC, 20 CD) patients [67]. Two studies assess quality-of-life (QoL) parameters and psychological states (anxiety, depression) before and after Ramadan. There was no correlation between Ramadan fasting and QoL. The mean score for anxiety was lower after Ramadan in the female UC patients [65], but no difference was found in depression levels [65,67]. Also, no significant changes in serum C-reactive protein (CRP) and stool calprotectin levels in the UC and CD patients were found [67]. Two studies reported that Ramadan fasting does not have serious risks for patients with IBD [65,66]. In the third study, an increase in the disease activity indices was observed in the UC patients of an older age or with higher baseline fecal calprotectin levels [67]. However, Ramadan fasting is a specialized form of fasting and may not reflect normal TRE or ADF. Thus, we included the existing studies on IF in healthy people.

## 5. Effect of IF on Immune Parameters in Healthy Subjects

In recent years, 16:8 fasting has become increasingly popular [68]. Several studies have investigated its anti-inflammatory benefits (Table 2). TRE appears to significantly ameliorate CRP levels in the serum blood of male participants after four weeks of fasting [68], but seems to have no significant impact after five weeks of fasting [69]. Otherwise, the inflammatory markers IGF-1, IL-6, TNF-α, and IL-1ß were significantly reduced whether the study lasted for two months or twelve months [70,71]. The comparison of early TRE (6 a.m.–3 p.m.) (eTRE) and mid-day TRE (11 a.m.–8 p.m.) (mTRE) revealed a significant reduction in TNF-α and IL-8 in the serum of the participants who took part in the eTRE, but not in the serum of participants from the mTRE group [69]. In contrast, in two TRE studies with three meals at 1 p.m., 4 p.m., and 8 p.m. corresponding to “mid-day TRE”, a significant reduction in the inflammatory markers IGF-1, IL-6, TNF-α, and IL-1β was found [70,71]. Microbial diversity was found to be positively influenced by TRE [69,72,73] because of the higher relative abundance of short chain fatty acid producers Prevotellaceae and Bacteroidetes in the TRE group [69,72,73]. Since Firmicutes was more abundant in the non-TRE group, the ratio between Bacteroidetes and Firmicutes indicates that TRE leads to a healthier gut microbiota [72]. Twelve hours of fasting for two days a week significantly decreased the number of white blood cells, lymphocytes, and neutrophils in the blood of young (25) and older men (52). CD4 cells were only increased in the older men [74]. Moreover, two studies analyzed the influence of ADF on women and men [75,76]. No differences in white blood cells, neutrophils, lymphocytes, monocytes, CD4 and CD8 cells, and CRP were found in the study that involved fasting for a month [75]. In the other study, in which the participants fasted for 3 months, significantly different CRP levels were observed [76]. The effect of TRE on clock gene expression was investigated in two studies [69,72]. TRE significantly increased the mRNA expression of the brain and muscle ARNT-like protein (BMAL)-1 and the circadian locomotor output cycle kaput (CLOCK), as well as sirtuin1 (SIRT1) gene expression. Circadian oscillations are important in protecting against metabolic disorders [72]. Again, differences were found between the early TRE and mid-day TRE groups. While eTRE significantly increased the amplitude of BMAL-1, period (PER) 2, and SIRT1 expression, mTRE also significantly increased the amplitude of PER2 expression, but significantly decreased the amplitude of PER1 gene expression [69].

## 6. Concluding Remarks

The positive influence of 16:8 fasting in humans seems to depend on the number of uninterrupted fasting days and possibly on the time of day when the feeding takes place. Concerning ADF, the number of continuous fasting days seems to be the decisive factor. The studies on Ramadan fasting showed almost no effect, but this is a very specific type of fasting which cannot be equated with TRE or ADF. However, TRE and ADF showed anti-inflammatory effects in healthy people. For these individuals, CRP levels and inflammatory markers were significantly reduced. Their microbiota was positively influenced, and circadian oscillation improved. Although the anti-inflammatory effect of TRE and ADF in humans depended on the number of continuous fasting days, the studies on mice and rats in which colitis was induced showed these improvements regardless of the duration of fasting. Histological scores, inflammatory markers, oxidative stress markers, and microbiota composition were improved by TRF. ADF also improved histological markers and inflammatory markers and even appeared to have a greater impact on histological markers. Furthermore, ADF improved small intestine barrier function and the relative abundance of colitis-associated bacteria. Despite the beneficial effects of TRE/TRF and ADF on human health and colitis in animal models, studies investigating the impact of TRE and ADF on IBD in humans are still missing. Many patients with IBD avoid certain foods, although avoiding self-prescribed foods does not prevent relapse. However, such diets can have a negative impact on mental health, quality of life, nutritional status, and the risk of malnutrition. For this reason, it is important to investigate the nutritional management of IBD patients in future human studies.

## Figures and Tables

**Table 1:** Effects of fasting on DAI, histology, inflammation, barriers, and oxidative stress in colitis in animal models.

Reference Year	Colitis Models	Settings	Fasting Models	DAI and Histology	Inflammation and Barrier Markers	Oxidative Stress
Sävendahl et al. 1997 [56]	5% DSS for 5 d	5 d n = 15/group	48 h fasting (3–5 d of DSS treatment)	DAI ↓ (colon)	*Il-1β* ↓^†^, *Igf-1* ↓^†^ (colon)	
Okada et al. 2017 [57]	3.5% DSS for 5 d	9 d n = 6/group	36 h fasting (6–8 d after DSS treatment)	crypts number ↑ (colon)	*Il-1β* ↓^†^, *Il-17* ↓^†^ (colon)	
Ige et al. 2020 [62]	1 × 1 mL 6% AA/ 100 g BW	11 d n = 6/group	TRF (10 d) 21.5 h fasting/d (after induction with AA)		TNF-α ↓^††^	GSH ↓^††^
Shahhat et al. 2020 [63]	3% AA for 7 d	28 d n = 7/group	TRF (3 w) 12/16/20 h fasting/d (after induction with AA)	hist. score ↓ (colon)	IL-1β ↓^††^, IL-6 ↓^††^, IL-8 ↓^††^	GSH ↑^††^
Song et al. 2022 [58]	2.5% DSS for 3 cycles of 5 d of DSS and 9 d of water	42 d n = 8/group	TRF (14 d) 18 h fasting for 7 d during DSS treatment for 2 cycles)	DAI ↓ hist. score ↓, crypts numbers ↑ (colon)	Infiltration of leukocytes and macrophages ↓ (colon), CD4^+^ T cells ↓ (PB and MLN), CD4^+^ CD25^+^ T cells ↑ (MLN)	
Zhang et al. 2020 [59]	2% DSS for 2 cycles of 6 d of DSS and 12 d of water	36 d n = 12/group	TRF (36 d) 16 h fasting/d (during DSS treatment)	DAI ↓ hist. score ↓, colon length ↑, goblet cells number ↑ (colon)	*Tnf-α* ↓^†^, *Il-1β* ↓^†^, *Il-6* ↓^†^, *Ifn-γ* ↓^†^ *Muc-2* ↑^†^, *Claudin-1* ↑^†^, *Zo-1* ↑^†^, *Occludin* ↑^†^ TNF-α ↓^††^, IL-1β ↓^††^ (colon)	GSSG ↓, GSH ↑ (cortex)
Wu et al. 2022 [60]	2% DSS for 7 d	3 w n = 8/group	ADF (2 w before DSS treatment)	DAI ↓ hist. score ↓, colon length ↑	IL-1α ↓^††^, IL-6 ↓^††^, KC ↓^††^, G-CSF ↓^††^	
Hadžić et al. 2024 [61]	0.5% DSS for 4 w and 6 cycles of 3 d with and 4 d without DSS	10 w n = 7/group	ADF (6 w after DSS treatment for 4 w)	villi length ↑ (jejunum)	*Tnf-α* ↓^†^, *Tgf-β1* ↓^†^, *Il-1β* ↓^†^, *Nod-2* ↓^†^ (colon)	

^†^ = mRNA expression; ^††^ = serum protein levels; ↑ = significant upregulation; ↓ = significant downregulation; *p* < 0.05 compared to controls (DSS-AL group); AA = acetic acid; ADF = alternate day fasting; BW = body weight; CD = cluster of differentiation; d = days; DAI = disease activity index; DSS = dextran sulfate sodium; G-CSF = granulocyte colony-stimulating factor; GSH = glutathione; GSSG = oxidized glutathione; hist. = histological; IGF = insulin-like growth factor; IL = interleukin; KC = keratinocyte-derived chemokine; Muc = mucin; MLN = mesenteric lymph nodes; PB = peripheral blood; TNF = tumor necrosis factor; TGF = transforming growth factor; TRF = time restricted feeding; w = weeks.

**Table 2:** Effects of fasting on humans (RCT studies).

Reference	Fasting Models	Participants	Immune Parameters in Serum	Microbiota	Clock Genes
Gasmi et al. 2018 [74]	TRE (12 w) 2 d fasting 12 h fasting/w (fasting on mon and thu)	men; n = 20; 26 y (TRE: 10, AL: 10)	WBCs ↓*, neutro ↓*, eosino ↔, baso ↔, mono ↔, lympho ↓#, CD4 ↑#, CD8 ↔		
		men; n = 20; 53 y (TRE: 10, AL: 10)	WBCs ↓*, neutro ↓*, eosino ↔, baso ↔, mono ↔, lympho ↓#, CD4 ↔, CD8 ↔		
McAllister et al. 2020 [68]	TRE (4 w) 16 h fasting/d	men; n = 22; 22 y (TRE: 10, AL: 12)	CRP ↓#		
Zeb et al. 2020 [72]	TRE (25 d) 16 h fasting/d	men; n = 80; (TRE: 56, AL: 24)	IL-1β ↔^†^, TNF-α ↔^†^	relative abundance: Bacteroidetes ↑#, Prevotellaceae ↑#	*BMAL-1* ↑#^†^, *CLOCK* ↑*#^†^, *SIRT1* ↑*#^†^
Zeb et al. 2020 [73]	TRE (25 d) 16 h fasting/d	men; n = 30; 18–38 y (TRE: 15, AL: 15)		relative abundance: Bacteroidetes ↑#, Prevotellaceae ↑#	
Moro et al. 2021 [71]	TRE (12 mo) 16 h fasting/d	healthy participants; n = 20; (TRE: 10, AL: 10)	IGF-1 ↓*#, IL-6 ↓*, TNF-α ↓*, IL-1ß ↓*		
Moro et al. 2016 [70]	TRE (8 w) 16 h fasting/d	men (trained); n = 34; 29 y (TRE: 17, AL: 17)	IGF-1 ↓*#, IL-6↓*#, TNF-α ↓*#, IL-1ß↓*#		
Xie et al. 2022 [69]	eTRE (5 w) 16 h fasting/d 6 a.m.–3 p.m.	women and men; n = 42; 29 y (TRE: 28, AL: 14)	TNF-α ↓, IL-8 ↓, CRP ↔	microbial diversity ↑	*BMAL-1 ↑*^†^, *PER2 ↑*^†^*SIRT1* ↑^†^ (amplitude)
	mTRE (5 w) 16 h fasting/d 11 a.m.–8 p.m.	women and men; n = 40; 29 y (TRE: 26, AL: 14)	CRP ↔		*PER2 ↑*^†^, *PER1 ↓*^†^ (amplitude)
Varady et al. 2013 [76]	ADF (12 w)	women and men; n = 30; 48 y (ADF: 15, AL: 15)	CRP ↓#		
Stekovic et al. 2019 [75]	ADF (4 w)	healthy participants; n = 90; 25 y (ADF: 30, AL: 60)	WBCs ↔, lympho ↔, mono ↔, CD4 ↔, CD8 ↔, B cell ↔, CRP ↔	β-hydroxy-butyrate ↔	

Age is presented as mean; ↑ = significant upregulation; ↓ = significant downregulation; ↔ = no significant change; * = significant difference (*p* < 0.05) from before and after TRE intervention; # = significant difference (*p* < 0.05) between AL and fasting; † = mRNA expression; ADF = alternate day fasting; AL = ad libitum; baso = basophils; BMAL= brain and muscle ARNT-like protein; CLOCK = circadian locomotor output cycle kaput; CD = cluster of differentiation; CRP = c-reactive protein; e = early, eosino = eosinophils; IGF = insulin-like growth factor; IL = interleukin; lympho = lymphocytes; m = mid-day; mo = months; mon = Monday; mono = monocytes; neutro = neutrophils; Per = period; SIRT = sirtuin; thu = Thursday; TNF = tumor necrosis factor; TRE = time-restricted eating; WBCs = white blood cells; y = years.
